# ﻿New species and records of *Phaeobotryon* (Botryosphaeriales, Botryosphaeriaceae) from *Larix* in China

**DOI:** 10.3897/mycokeys.112.139053

**Published:** 2025-01-06

**Authors:** Yeting Zhu, Yingmei Liang, Cheng Peng

**Affiliations:** 1 The Key Laboratory for Silviculture and Conservation of the Ministry of Education, Beijing Forestry University, Beijing 100083, China Beijing Forestry University Beijing China; 2 Museum of Beijing Forestry University, Beijing Forestry University, Beijing 100083, China Beijing Forestry University Beijing China

**Keywords:** Ascomycota, molecular phylogeny, morphology, taxonomy

## Abstract

During the fungal investigations of *Larix* hosts in China, ten isolates of *Phaeobotryon* were obtained from dead and dying branches. Morphological characteristics and phylogenetic analyses of combined ITS, LSU, and *tef1-α* loci revealed the presence of two new species, *P.laricinum* and *P.longiparaphysium*, as well as two new host records for *P.aplosporum* and *P.rhois* from *L.olgensis*. In this study, we provide descriptions and illustrations of these species, thereby enriching the diversity within the *Phaeobotryon* taxa.

## ﻿Introduction

*Phaeobotryon* is a monophyletic genus typed with *P.cercidis*, belonging to Dothideomycetes, Botryosphaeriales, Botryosphaeriaceae (Phillips et al. 2008; Liu et al. 2012). This genus was established by Theissen and Sydow (1915) to classify *Dothideacercidis*, which produces light brown, two-septate ascospores. Subsequently, *Phaeobotryon* was considered a synonym of *Botryosphaeria* based on the morphology of the sexual morph (Von Arx and Müller 1954, 1975). However, more recent morphological and phylogenetic data indicated that *Phaeobotryon* constitutes a distinct lineage within the Botryosphaeriaceae, characterized by the 2-septate, brown ascospores with an apiculus at each end (Phillips et al. 2008, 2013; Liu et al. 2012; Zhang et al. 2021).

As of now, 16 epithets of *Phaeobotryon* have been listed in Index Fungorum (www.indexfungorum.org; accessed on 10 October 2024). Of these, *P.disruptum*, *P.euganeum*, and *P.visci* have been removed from *Phaeobotryon* and reassigned to their respective genera (Von Arx and Müller 1954; Barr 2001). Consequently, 13 species are accepted within the genus, of which *P.cercidis* and *P.quercicola* lack available DNA sequence data. Species of *Phaeobotryon* have primarily been discovered in Asia, Europe, and North America. Members of this genus exhibit a wide range of hosts, encompassing 24 genera, including three genera of gymnosperms: *Calocedrus*, *Juniperus*, and *Platycladus* (Phillips et al. 2005, 2008; Abdollahzadeh et al. 2009; Fan et al. 2015; Daranagama et al. 2016; Zhu et al. 2018; Pan et al. 2019; Wijayawardene et al. 2021; Zhang et al. 2021; Hattori and Masuya 2023; Lin et al. 2023; Peng et al. 2023; Wu et al. 2024; Zhou et al. 2024). Until now, *Phaeobotryon* has not been reported to inhabit *Larix*.

The number of *Phaeobotryon* species has increased rapidly in recent years, with nine of the 13 species published since 2015, seven of which have been described in China. This trend suggests that the genus exhibits significant diversity within the country. During our investigation of fungal diseases affecting *Larix* in China, four *Phaeobotryon* species were collected from *L.gmelinii*, *L.olgensis*, and L.gmeliniivar.principis-rupprechtii in Heilongjiang, Jilin, and Hebei Provinces, respectively. This study used phylogenetic analysis and morphological comparisons to describe new species and document new host records, thereby enriching the fungal taxa within *Phaeobotryon*.

## ﻿Materials and methods

### ﻿Fungal isolation

Fresh specimens were collected from *Larixgmelinii*, *L.olgensis*, and L.gmeliniivar.principis-rupprechtii in Heilongjiang, Jilin, and Hebei Provinces, respectively. The specimens were carefully packed in kraft paper bags and transported to the laboratory for fungal isolation. Isolates were obtained using the single spore isolation method as described by Chomnunti et al. (2014). Following incubation at 25 °C, single germinating conidia were transferred to fresh plates of PDA. The cultures have been deposited in the China Forestry Culture Collection Center (**CFCC**), while the specimens are stored in the Museum of Beijing Forestry University (**BJFC**).

### ﻿Morphology

Morphological observations were conducted on conidiomata produced on infected plant tissues. The conidiomata were manually sectioned using a double-edged blade and examined under a dissecting microscope for both macroscopic and microscopic characterization. The structure and size of the conidiomata were imaged with a Leica stereomicroscope (M205) (Leica Microsystems, Wetzlar, Germany). Additionally, conidia and other microstructures were randomly selected for observation using a Nikon Eclipse 80i microscope (Nikon Corporation, Tokyo, Japan), which was equipped with a Nikon digital sight DSRi2 high-definition color camera featuring differential interference contrast (DIC). More than 50 conidia were measured per species, and 30 measurements were taken of other morphological structures. Colony characteristics, including color and texture on PDA at 25 °C, were observed and recorded over 14 days. The colony colors were determined based on the color charts of Rayner (1970).

### ﻿DNA extraction, amplification, and sequencing

DNA was extracted using the modified CTAB method (Doyle and Doyle 1990) and stored at -20 °C. To confirm species identity, the internal transcribed spacer (ITS) region of all isolates was sequenced. The resulting sequences were subsequently compared with those in GenBank using the BLAST tool (https://blast.ncbi.nlm.nih.gov/Blast.cgi). Following genus-level confirmation, additional loci, including the nuclear ribosomal large subunit (LSU) and partial translation elongation factor 1-alpha (*tef1-α*), were amplified. The PCR mixture for all regions comprised 1 µL of DNA template, 1 µL of each 10 µM primer, 10 µL of T5 Super PCR Mix (which contains Taq polymerase, dNTP, and Mg^2+^; Beijing TisingKe Biotech Co., Ltd., Beijing, China), and 7 µL of sterile water. The primers and PCR conditions are detailed in Table 1. PCR products were electrophoresed in a 1% agarose gel and subsequently sequenced by Beijing TisingKe Biotech Co., Ltd. (Beijing, China). The forward and reverse reads were edited and assembled using Seqman v.7.1.0 software.

**Table 1.** Loci, PCR primers, protocols, and references used in this study.

Locus	Primers	Thermal cycles	Reference
ITS	ITS1/ITS4	(95 °C: 30 s, 51 °C: 30 s, 72 °C: 1 min) × 35 cycles	White et al. (1990)
LSU	LR0R/LR5	(95 °C: 45 s, 55 °C: 45 s, 72 °C: 1 min) × 35 cycles	Vilgalys and Hester (1990), Rehner and Samuels (1994)
*tef1-α*	EF1-728F/EF1-986R(EF1-1567R)	(95 °C: 15 s, 55 °C: 20 s, 72 °C: 1 min) × 35 cycles	Carbone and Kohn (1999), Rehner and Buckley (2005)

### ﻿Phylogenetic analyses

All sequences generated in this study were submitted to GenBank, and reference sequences from known species were downloaded from the National Center for Biotechnology Information (NCBI; https://www.ncbi.nlm.nih.gov) to construct the phylogenetic analysis (Table 2). The individual datasets for each gene region were aligned separately using MAFFT v. 6.0 (Katoh and Standley 2013) and subsequently trimmed at both terminal ends in MEGA v. 6.0 (Tamura et al. 2013). MEGA v. 6 software was utilized to align and edit the sequences, with *Lasiodiplodiatheobromae* (CBS 164.96) selected as the outgroup. Multi-gene phylogenetic analyses employing maximum parsimony (MP), maximum likelihood (ML), and Bayesian inference (BI) were conducted using PAUP v. 4.0b10, raxmlGUI v. 1.5b1, and MrBayes v. 3.1.2 software, respectively. The resulting phylogenetic tree was visualized using Figtree v. 1.4.0 and modified with AI CS v. 5. The support rate of MP and ML analysis was greater than 50%, and the posterior probability of BI analysis was greater than 0.9 as presented in the tree.

**Table 2.** Taxa used for molecular phylogenetic analyses and their GenBank accession numbers. (T) = ex-type strains. Isolates in this study are shown in bold.

Species	Strain	Host	Origin	GenBank accession numbers		
				ITS	LSU	*tef1-α*
*Alanphillipsiaaloeicola*	CBS 138896 = CPC 23674^T^	*Aloe* sp.	South Africa	Kp004444	Kp004472	Mt592027
*A.aloeigena*	CBS 136408 = CPC 21286^T^	*Aloemelanocantha*	South Africa	Kf777137	Kf777193	–
*A.aloes*	CBS 136410 = CPC 21298^T^	*Aloedichotoma*	South Africa	Kf777138	Kf777194	–
*A.aloetica*	CBS 136409 = CPC 21110, 21109^T^	*Aloe* sp.	South Africa	Kf777139	Kf777195	Mt592028
*A.euphorbiae*	CBS 136411 = CPC 21629, 21628^T^	*Euphorbia* sp.	South Africa	Kf777140	Kf777196	Mt592029
*Barriopsisarchontophoenicis*	MFLUCC 14-1164^T^	*Archontophoenixalexandrae*	Thailand	Kx235306	Kx235307	–
*B.iraniana*	CBS 124698 = IRAN 1448C^T^	*Mangiferaindica*	Iran	Fj919663	Kf766318	Fj919652
*B.iraniana*	CBS 124699 = IRAN 1449C	*Olea* sp.	Iran	Fj919665	Kx464241	Fj919654
*B.stevensiana*	CBS 174.26^T^	*Citrus* sp.	Cuba	Eu673330	Dq377857	Eu673296
*B.tectonae*	CBS 137786 = MFLUCC 12-0381 = CMW 40687^T^	*Tectonagrandis*	Thailand	Kj556515	Mh878606	Kj556516
*B.thailandica*	MFLUCC 14-1190 = KUMCC 16-0185^T^	*Tectonagrandis*	Thailand	Ky115675	–	Ky115676
*Lasiodiplodiatheobromae*	CBS 164.96^T^	Fruit along coral reef coast	Papua	Ay640255	Eu673253	Ku696383
*Oblongocollomycesvariabilis*	CBS 121774 = CMW 25419 = CAMS 1174^T^	*Acaciakarroo*	Namibia	Eu101312	Kx464536	Eu101357
*O.variabilis*	CBS 121775 = CMW 25421 = CAMS 1176	*Aplosporellakaroo*	Namibia	Eu101314	Mt587319	Eu101359
*O.variabilis*	CBS 121776 = CMW 25422 = CAMS 1177	*Acaciamellifera*	South Afri	Eu101326	Kx464537	Eu101371
*Phaeobotryonaplosporum*	CFCC 53773	*Syzygiumaromaticum*	China	Mn215835	Mn215870	Mn205995
*P.aplosporum*	CFCC 53774	*Syzygiumaromaticum*	China	Mn215836	Mn215871	Mn205996
*P.aplosporum*	CFCC 53775^T^	*Rhustyphina*	China	Mn215837	Mn215872	–
*P.aplosporum*	CFCC 53776	*Rhustyphina*	China	Mn215838	Mn215873	Mn205997
***P.aplosporum***	**CFCC 70810**	***Larixolgensis***	**China**	**Pp960186**	**Pp960196**	**Pq046939**
***P.aplosporum***	**CFCC 70811**	***Larixolgensis***	**China**	**Pp960187**	**Pp960197**	**Pq046940**
*P.cupressi*	CBS 124700 = IRAN 1455C^T^	*Cupressussemipervirens*	Iran	Fj919672	Kx464538	Fj919661
*P.cupressi*	CBS 124701 = IRAN 1458C	*Cupressussemipervirens*	Iran	Fj919671	Kx464539	Fj919660
*P.cupressi*	IRAN 1454C	*Cupressussemipervirens*	Iran	Fj919673	–	Fj919662
*P.fraxini*	CFCC 70762^T^	*Fraxinuschinensis*	China	Pp188527	Pp177348	–
*P.fraxini*	CFCC 70763	*Fraxinuschinensis*	China	Pp188528	Pp177349	–
*P.juniperi*	JU 001^T^	*Juniperusformosana*	China	Op941637	Op941644	Op948218
*P.juniperi*	JU 005	*Juniperusformosana*	China	Op941638	Op941645	Op948219
*P.juniperi*	JU 007	*Juniperusformosana*	China	Op941639	Op941646	Op948220
***P.laricinum***	**CFCC 70804**	***Larixolgensis***	**China**	**Pp960188**	**Pp960198**	**Pq046941**
***P.laricinum***	**CFCC 70805^T^**	***Larixolgensis***	**China**	**Pp960189**	**Pp960199**	**Pq046942**
***P.laricinum***	**CFCC 70806**	***Larixgmelinii***	**China**	**Pp960190**	**Pp960200**	**Pq046943**
***P.longiparaphysium***	**CFCC 70807^T^**	**Larixgmeliniivar.principis-rupprechtii**	**China**	**Pp960193**	**Pp960203**	**Pq046946**
***P.longiparaphysium***	**CFCC 70808**	**Larixgmeliniivar.principis-rupprechtii**	**China**	**Pp960194**	**Pp960204**	**Pq046947**
***P.longiparaphysium***	**CFCC 70809**	***Larixolgensis***	**China**	**Pp960195**	**Pp960205**	**Pq046948**
*P.mamane*	CBS 122980 = CPC 12440^T^	*Sophorachrysophylla*	USA	Eu673332	Eu673248	Eu673298
*P.mamane*	CPC 12442	*Sophorachrysophylla*	USA	Eu673333	Dq377899	Eu673299
*P.mamane*	CPC 12443	*Sophorachrysophylla*	USA	Eu673334	Eu673249	Eu673300
*P.negundinis*	CAA 797	*Acernegundo*	Russia	Kx061513	–	Kx061507
*P.negundinis*	CAA 798	*Ligustrumvulgare*	Russia	Kx061514	–	Kx061508
*P.negundinis*	CAA 799	*Forsythiaintermedia*	Russia	Kx061515	–	Kx061509
*P.negundinis*	CPC 33388	Dead stem	Ukraine	Mt587543	Mt587324	Mt592277
*P.negundinis*	CPC 34752	*Acernegundo*	Ukraine	Mt587544	Mt587325	Mt592278
*P.negundinis*	MFLUCC 15-0436^T^	*Acernegundo*	Russia	Ku820970	–	Ku853997
*P.platycladi*	CFCC 58799^T^	*Platycladusorientalis*	China	Oq651172	Oq652543	Oq692930
*P.platycladi*	CFCC 58800	*Platycladusorientalis*	China	Oq651173	Oq652544	Oq692931
*P.rhoinum*	CFCC 52449	*Rhustyphina*	China	Mh133923	Mh133940	Mh133957
*P.rhoinum*	CFCC 52450^T^	*Rhustyphina*	China	Mh133924	Mh133941	Mh133958
*P.rhoinum*	CFCC 52451	*Rhustyphina*	China	Mh133925	Mh133942	Mh133959
*P.rhois*	CFCC 89662 = CCTCC AF2014017^T^	*Rhustyphina*	China	Km030584	Km030591	Km030598
*P.rhois*	CFCC 89663 = CCTCC AF2014016	*Rhustyphina*	China	Km030585	Km030592	Km030599
*P.rhois*	CFCC 58679	Populusalbavar.pyramidalis	China	Oq651171	Oq652542	Oq692929
*P.rhois*	CFCC 52448	*Rhustyphina*	China	Mh133922	Mh133939	Mh133956
*P.rhois*	CFCC 53777	*Platycladusorientalis*	China	Mn215839	Mn215874	
*P.rhois*	CFCC 53779	*Rhamnusdahuricus*	China	Mn215841	Mn215876	Mn205999
*P.rhois*	CFCC 53780	*Dioscoreanipponica*	China	Mn215842	Mn215877	Mn206000
***P.rhois***	**CFCC 70812**	***Larixolgensis***	**China**	**Pp960191**	**Pp960201**	**Pq046944**
***P.rhois***	**CFCC 70813**	***Larixolgensis***	**China**	**Pp960192**	**Pp960202**	**Pq046945**
*P.spiraeae*	CFCC 53925^T^	*Spiraeasalicifolia*	China	Om049420	Om049432	–
*P.spiraeae*	CFCC 53926	*Spiraeasalicifolia*	China	Om049421	Om049433	–
*P.spiraeae*	CFCC 53927	*Spiraeasalicifolia*	China	Om049422	Om049434	–
*P.ulmi*	94-13	*Ulmuspumila*	USA	Af243398	–	–
*P.ulmi*	CBS 114123 = UPSC 2552	*Ulmusglabra*	Sweden	Mt587539	Mt587320	Mt592273
*P.ulmi*	CBS 138854 = CPC 24264^T^	*Ulmusleavis*	Germany	Mt587540	Mt587321	Mt592274
*P.ulmi*	CBS 123.30 = ATCC 24443 = DSM 2491 = MUCL 10057	*Ulmus* sp.	USA	Kx464232	Dq377861	Kx464766
*P.ulmi*	CBS 174.63	*Ulmusglabra*	Finland	Mt587541	Mt587322	Mt592275
*P.ulmi*	CMH 299	House dust	USA	Kf800390	–	–
*P.ulmi*	PB 11f	*Ulmusglabra*	Poland	Mk134682	–	–
*Sphaeropsiscitrigena*	ICMP 16812^T^	*Citrussinensis*	New Zealand	Eu673328	Eu673246	Eu673294
*S.citrigena*	ICMP 16818	*Citrussinensis*	New Zealand	Eu673329	Eu673247	Eu673295
*S.eucalypticola*	CBS 133993 = MFLUCC 11-0579 = CPC 21560 = BT 021^T^	*Eucalyptus* sp.	Thailand	Jx646802	Jx646819	Jx646867
*S.eucalypticola*	MFLUCC 11-0654	*Eucalyptus* sp.	Thailand	Jx646803	Jx646820	Jx646868
*S.porosa*	CBS 110496 = CPC 5132 = JM 29 = STE-U 5132^T^	*Vitisvinifera*	South Africa	Ay343379	Dq377894	Ay343340
*S.porosa*	CBS 110574 = STE-U 5046	*Vitisvinifera*	South Africa	Ay343378	Dq377895	Ay343339
*S.visci*	CBS 100163 = 12273	*Viscumalbum*	Luxembourg	Eu673324	Dq377870	Eu673292
*S.visci*	CBS 122526 = CAP 350^T^	*Viscumalbum*	Ukraine	Eu673326	Kx464550	–
*S.visci*	CBS 122527 = CAP 349	*Viscumalbum*	Ukraine	Eu673327	Kx464551	Kx464776
*S.visci*	CBS 186.97	*Viscumalbum*	Germany	Eu673325	Dq377868	Eu673293
*S.visci*	CPC 33386	Dead leaf	Ukraine	Mt587557	Mt587326	Mt592305
*S.visci*	CPC 35421	*Viscumalbum*	Germany	Mt587558	Mt587327	–
*S.visci*	CPC 35525	*Eucalyptusgrandis*	Australia	Mt587559	Mt587328	Mt592306

## ﻿Results

### ﻿Phylogenetic analysis

**Figure 1.**
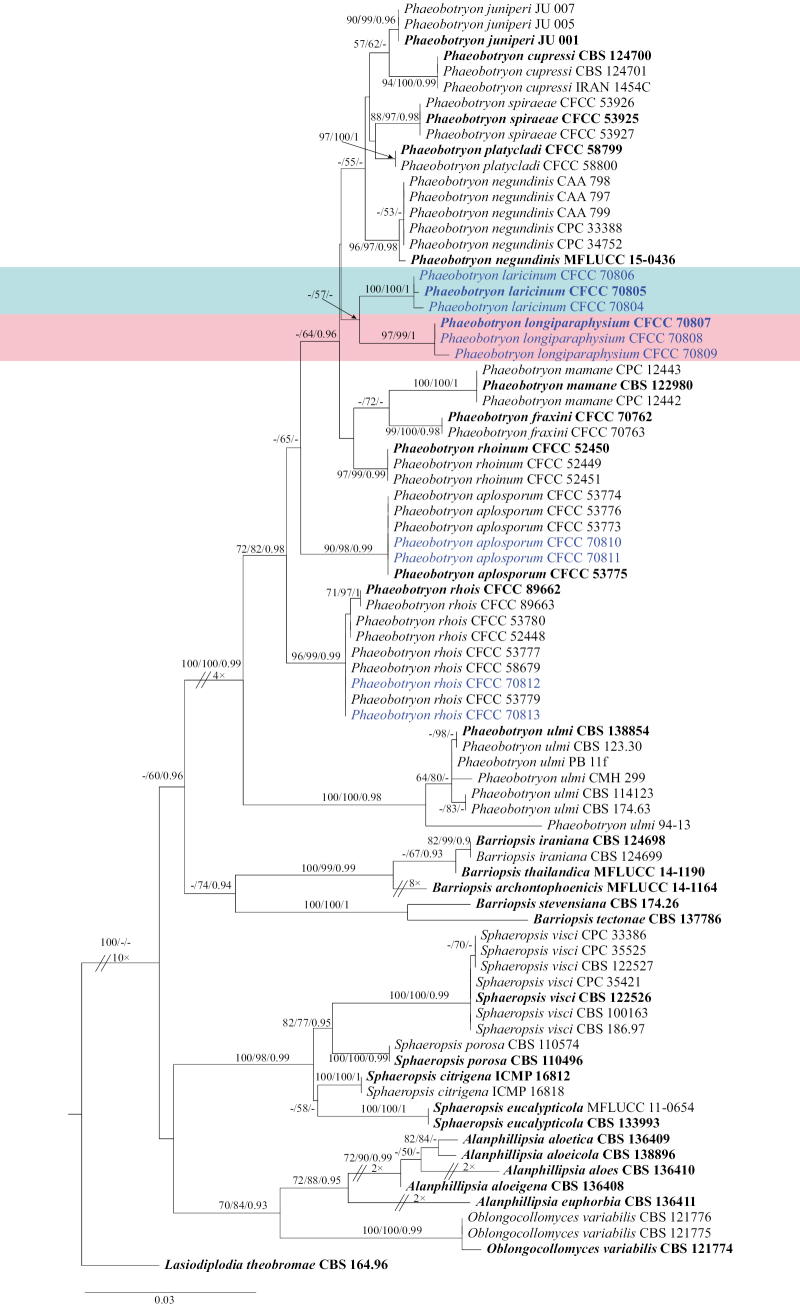
Phylogenetic tree inferred from ML analysis based on combined ITS, LSU, and *tef1-α* sequence data of *Phaeobotryon* isolates. The tree was rooted in *Lasiodiplodiatheobromae* (CBS 164.96). The MP, ML (≥ 50%), and BI (≥ 0.9) bootstrap values are given at nodes (MP/ML/BI). Isolates from this study are marked in blue, ex-type strains are marked in bold, and new species are in a colored font.

The gene loci of ITS, LSU, and *tef1-α* were combined and analyzed to infer the phylogenetic placement of our isolates in the genus *Phaeobotryon*. The dataset includes 81 sequences; of these, *Lasiodiplodiatheobromae* (CBS 164.96) was set as the outgroup taxon. The combined dataset after alignment consisted of 1,744 characters, including 508 characters in ITS, 757 characters in LSU, and 469 characters in *tef1-α* gaps that were included in the phylogenetic analysis. In the alignment, 1,346 characters are constant, 120 variable characters are parsimony-uninformative, and 120 characters are parsimony-informative. In ML analysis based on the combined gene dataset, the matrix had 488 distinct alignment patterns. Estimated base frequencies are as follows: A = 0.226764, C = 0.257594, G = 0.287105, T = 0.228538; substitution rates: AC = 1.111156, AG = 2.606477, AT = 0.720936, CG = 1.166284, CT = 5.223120, GT = 1.000000. Trees from Bayesian analyses and MP were identical to that of the ML tree shown (Fig. 1). Isolates CFCC 70804, CFCC 70805, and CFCC 70806, as well as isolates CFCC 70807, CFCC 70808, and CFCC 70809, are clustered into separate lineages and are designated as two new species. Isolates CFCC 70810 and CFCC 70811 are grouped with *P.aplosporum*, while isolates CFCC 70812 and CFCC 70813 are grouped with *P.rhois*, thus designated as species *P.aplosporum* and *P.rhois*, respectively.

### ﻿Taxonomy

#### ﻿ Phaeobotryon laricinum

Taxon classificationFungiBotryosphaerialesBotryosphaeriaceae

Y.T. Zhu & Y.M. Liang sp. nov.

E95946F3-E0F6-5818-BFD6-8B0E92B63C6C

 854522

Fig. 2

##### Etymology.

Named after the host genus on which it was collected, *Larix*.

##### Descriptions.

***Sexual morph***: Not observed. ***Asexual morph***: Conidiomata pycnidial, scattered, immersed, or semi-immersed to erumpent from bark surface, globose to ovoid, unilocular, 365–820 µm diam. Disc black, 215–360 µm in diam. Ostioles single, central, 35–75 µm. Conidiophores reduced to conidiogenous cells. Paraphyses present, hyaline, thin-walled, arising from the conidiogenous layer, extending above the level of developing conidia, tip rounded, aseptate, up to 60.5 × 2.5 µm. Conidiogenous cells hyaline, smooth, thin-walled, holoblastic, cylindrical, phialidic, proliferating internally with visible periclinal thickening, 11.0–41.0 × 1.0–3.5 µm. Conidia initially hyaline, becoming brown with age, dark brown, aseptate, smooth with granular contents, guttulate, thick-walled, oblong to cylindrical, straight, both ends broadly rounded, 27.5–37.0 × 10.0–18.0 µm (av. ± S.D. = 32.2 ± 2.08 × 14.01 ± 1.77 µm), L/W = 2.3 ± 0.3.

**Figure 2.**
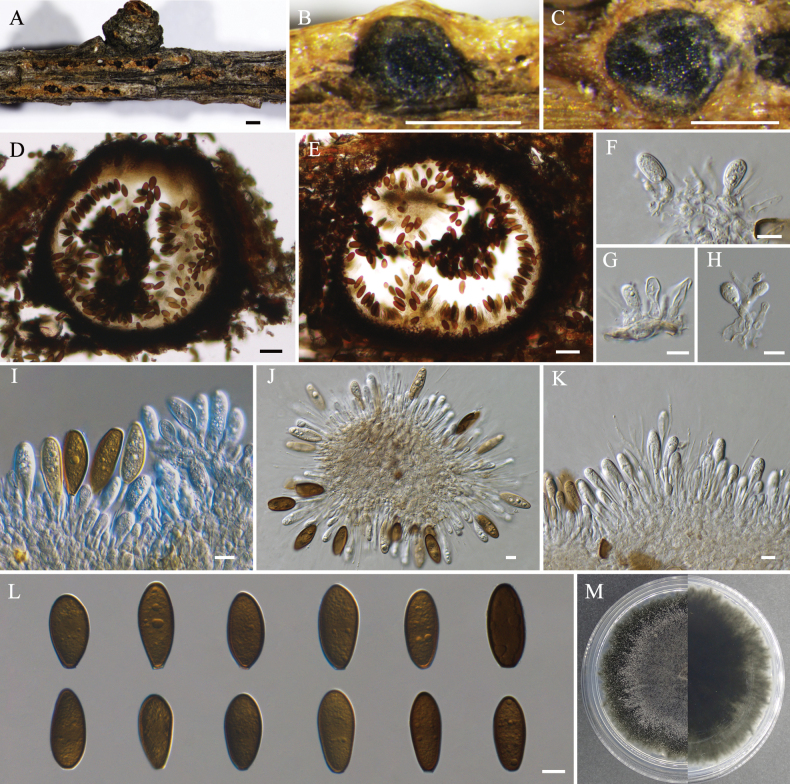
*Phaeobotryonlaricinum* (BJFC-S2370) **A** habit of conidiomata on twig **B, D** longitudinal section through a conidioma **C, E** transverse section of a conidioma **F–K** conidiogenous cells and conidia **L** conidia **M** colony on PDA after 14 days. Scale bars: 500 µm (**A–C**); 50 µm (**D, E**); 10 µm (**F–L**);

##### Culture characteristics.

Colonies on PDA flat, spreading, with flocculent mycelium and uneven edges, initially white, gradually turning greenish-grey from center, finally becoming black, covering 40–50 mm after 7 days at 25 °C.

##### Materials examined.

China • Jilin Province, Yanbian Korean Autonomous Prefecture, Yanji City, Maoershan National Forest (42°51'12.96"N, 129°28'24.06"E), alt. 297 m, on branches of *Larixolgensis*, 7, Sept, 2022, C. Peng, X.Y. Zhang (holotype BJFC-S2370, ex-holotype culture CFCC 70805; isotype BJFC-2371, ex-isotype culture CFCC 70806); China • Heilongjiang Province, Greater Khingan Mountains, Tahe County, Qixiashan Mountains (52°20'32.96"N, 124°41'48.27"E), alt. 456 m, on branches of *Larixgmelinii*, 10, Sept, 2021, R. Wang, W.T. Yu (BJFC-S2369, living culture CFCC 70804).

##### Notes.

*Phaeobotryon* currently comprises 13 species, all of which have reported asexual morphs except for *P.cercidis* (Phillips et al. 2005, 2008; Abdollahzadeh et al. 2009; Fan et al. 2015; Daranagama et al. 2016; Zhu et al. 2018; Pan et al. 2019; Wijayawardene et al. 2021; Zhang et al. 2021; Lin et al. 2023; Peng et al. 2023; Wu et al. 2024). However, *P.cercidis* has been reported on *Cerciscanadensis* in the USA (Phillips et al. 2008), revealing differences from *P.laricinum* in terms of both geographic region (China) and host (*Larix*). The new species can be distinguished from other known species based on conidial characteristics (Table 3). Specifically, *P.laricinum* conidia are aseptate and can be differentiated from other species in the genus, except for *P.negundinis*, *P.quercicola*, and *P.spiraeae*. Furthermore, they can be distinguished by conidial color (dark brown) from *P.quercicola* (hyaline). Additionally, the conidial size of *P.laricinum* (27.5–37 × 10–18 μm) is significantly larger than that of both *P.negundinis* (16–24.5 × 7.9–11.5 μm) and *P.spiraeae* (21–28.5 × 8.5–13.5 μm). Moreover, *P.laricinum* (L/W = 2.3 ± 0.3) can be distinguished by its larger conidial L/W ratio when compared to the new species *P.longiparaphysium* (L/W = 1.7 ± 0.2) (Table 3). Phylogenetically, *P.laricinum* is distinct from other *Phaeobotryon* species, which are grouped within a separate clade that receives high support (MP/ML/BI = 100/100/1) (Fig. 1). Therefore, *P.laricinum* is introduced as a novel species.

**Table 3.** Conidia comparison of species in *Phaeobotryon* (new species in bold).

Species	Septation	colour	size (μm)	Reference
*Phaeobotryonaplosporum*	aseptate	dark brick	15–21.5 × 5.5–7	Pan et al. 2019
*P.cercidis*	No record	No record	No record	Phillips et al. 2008
*P.cupressi*	1-septate	brown	19.8–30 × 10.2–17, L/W = 2 ± 0.3	Abdollahzadeh et al. 2009
*P.fraxini*	1-septate	brownish yellow to dark brown	13.0–20.0 × 7.0–10.0	Wu et al. 2024
*P.juniperi*	1-septate	dark brown	23–28.5 × 11.5–14	Peng et al. 2023
***P.laricinum***	**aseptate**	**dark brown**	**27.5–37 × 10–18, L/W = 2.3 ± 0.3**	**This study**
***P.longiparaphysium***	**aseptate**	**dark brown**	**24–36.5 × 15–20.5, L/W = 1.7 ± 0.2**	**This study**
*P.mamane*	1(–2)-septate	brown	30–43 × 12–16	Phillips et al. 2008, 2013
*P.negundinis*	aseptate	dark brown	16–24.5 × 7.9–11.5	Daranagama et al. 2016
*P.platycladi*	aseptate, rarely becoming 1-septate	initially hyaline	23.0–31.0 × 9.5–12.5	Lin et al. 2023
*P.quercicola*	aseptate	hyaline	24–38 × 11–21.2, L/W = 2.1	Phillips et al. 2005, 2008
*P.rhoinum*	1-septate	brown	18.5–21.5 × 7–9	Zhu et al. 2018
*P.rhois*	1-septate	brown	19–25 × 10–12	Fan et al. 2015
*P.spiraeae*	aseptate	dark brown	21–28.5 × 8.5–13.5	Wijayawardene et al. 2021
*P.ulmi*	1-septate	brown	26–34.5 × 15–20	Zhang et al. 2021

#### ﻿ Phaeobotryon longiparaphysium

Taxon classificationFungiBotryosphaerialesBotryosphaeriaceae

Y.T. Zhu & Y.M. Liang sp. nov.

8259383E-529F-53DE-B585-DED2BACD3328

 854523

Fig. 3

##### Etymology.

Named after the long paraphyses of conidiomata.

##### Descriptions.

***Sexual morph***: Not observed. ***Asexual morph***: Conidiomata pycnidial, scattered, immersed or semi-immersed, globose to ovoid, unilocular, 280–550 µm diam. Disc black, 180–330 µm in diam. Ostioles single, central, 65–115 µm. Paraphyses present, hyaline, thin-walled, arising from the conidiogenous layer, extending above the level of developing conidia, tip rounded, aseptate, up to 74.5 × 2.5 µm. Conidiophores reduced to conidiogenous cells. Conidiogenous cells hyaline, smooth, thin-walled, holoblastic, cylindrical, phialidic, proliferating internally with visible periclinal thickening, 9.5–29.0 × 1.0–4.0 µm. Conidia initially hyaline, becoming brown with age, dark brown, thick-walled, oval, with obtuse or gradually acute apex, rounded, gradually acute base, aseptate, 24.0–36.5 × 15.0–20.5 µm (av. ± S.D. = 31.69 ± 2.86 × 18.34 ± 1.01 µm), L/W = 1.7 ± 0.2.

**Figure 3.**
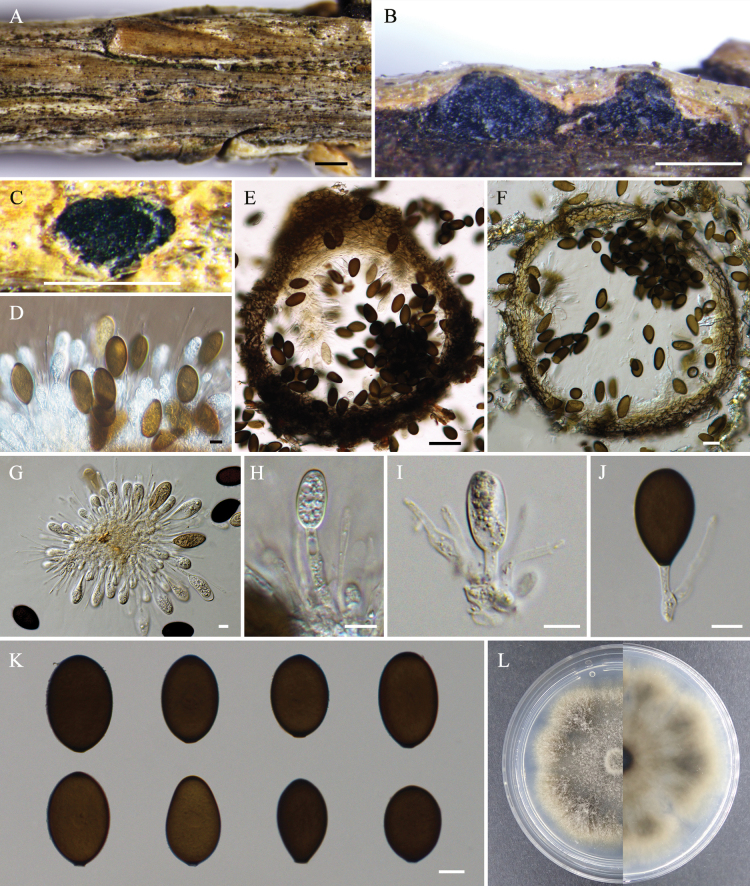
*Phaeobotryonlongiparaphysium* (BJFC-S2372) **A** habit of conidiomata on twig **B, E** longitudinal section through a conidioma **C, F** transverse section of a conidioma **D, G–J** conidiogenous cells and conidia **K** conidia **L** colony on PDA after 14 days. Scale bars: 500 µm (**A–C**); 50 µm (**E, F**); 10 µm (**D, G–K**).

##### Culture characteristics.

Colonies on PDA with aerial mycelium, thick and fluffy at the edge, margin with undulate and irregular, initially white, gradually turning brown, finally becoming black, covering 40–50 mm after 7 days at 25 °C.

##### Materials examined.

China • Hebei Province, Chengde City, Saihanba Forest Farm (42°28'37.08"N, 117°25'45.49"E), alt. 1657 m, on branches of Larixgmeliniivar.principis-rupprechtii, 10, Jul. 2023, C.M. Tian, C. Peng, S.J. Li, Y. Yuan, M.W. Zhang (holotype BJFC-S2372, ex-holotype cultures CFCC 70807, CFCC 70808). China • Jilin Province, Yanbian Korean Autonomous Prefecture, Yanji City, Maoershan National Forest (42°51'12.96"N, 129°28'24.06"E), alt. 297 m, on branches of *Larixolgensis*, 7, Sept, 2022, C. Peng, X.Y. Zhang (BJFC-S2373, living culture CFCC 70809).

##### Notes.

*Phaeobotryonlongiparaphysium* formed a distinct clade (MP/ML/BI = 97/99/1) in the multi-locus analyses and is sister to *P.laricinum* (Fig. 1). These two species can be distinguished based on the ITS, LSU, and *tef1-α* loci, with *P.longiparaphysium* exhibiting 29 bp differences from *P.laricinum* (5/535 in ITS, 5/780 in LSU, and 19/291 in *tef1-α*). Furthermore, *P.longiparaphysium* (L/W = 1.7 ± 0.2) can be distinguished from *P.laricinum* (L/W = 2.3 ± 0.3) by its smaller conidial L/W ratio (Table 3).

#### ﻿ Phaeobotryon aplosporum

Taxon classificationFungiBotryosphaerialesBotryosphaeriaceae

M. Pan & X.L. Fan, Mycol. Prog. 18(11): 1356 (2019)

5FDA8A85-C62C-5531-82D3-68B07338EE88

##### Descriptions.

See Pan et al. 2019.

##### Materials examined.

China • Jilin Province, Yanbian Korean Autonomous Prefecture, Yanji City, Maoershan National Forest (42°51'12.96"N, 129°28'24.06"E), alt. 297 m, on branches of *Larixolgensis*, 7, Sept, 2022, C. Peng, X.Y. Zhang (BJFC-S2375, living culture CFCC 70810, CFCC 70811).

##### Notes.

**Figure 4.**
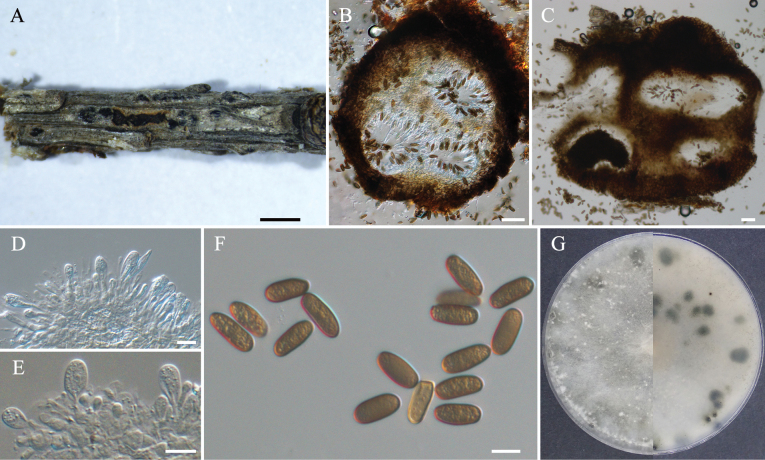
*Phaeobotryonaplosporum* (BJFC-S2375) **A** habit of conidiomata on twig **B** longitudinal section through a conidioma **C** transverse section of a conidioma **D, E** conidiogenous cells and conidia **F** conidia **G** colony on PDA after 7 days. Scale bars: 500 µm (**A**); 50 µm (**B, C**); 10 µm (**D–F**).

*Phaeobotryonaplosporum* was first identified in *Rhustyphina* and *Syzygiumaromaticum* (Pan et al. 2019). Since then, this species has also been reported in *Juglansmandshurica* (Lin et al. 2023), *Wisteriafloribunda*, *Malus* sp., and *Kerriajaponica* (Hattori and Masuya 2023). In this study, we observed the asexual morph of *P.aplosporum* (Fig. 4), which features unilocular conidiomata that differ from previously published descriptions for other hosts. It is known from previous literature reports that *P.quercicola* exhibits both unilocular conidiomata and multilocular conidiomata (Phillips et al. 2008), and *P.cupressi* mostly unilocular conidiomata on pine needles and mostly multilocular conidiomata on *Populus* twigs (Abdollahzadeh et al. 2009). This suggests that both unilocular and multilocular conidiomata may coexist within the same species in this genus. Additionally, the other characteristics of *P.aplosporum* in *L.olgensis* we observed (Fig. 4) are consistent with those noted from other hosts. Phylogenetically, the isolates CFCC 70810 and CFCC 70811 clustered within a clade with *P.aplosporum*, demonstrating high statistical support (MP/ML/BI = 90/98/0.99) (Fig. 1). Therefore, the isolates CFCC 70810 and CFCC 70811 are identified as *P.aplosporum*. This study extends the host range of *P.aplosporum* to include *L.olgensis*.

#### ﻿ Phaeobotryon rhois

Taxon classificationFungiBotryosphaerialesBotryosphaeriaceae

C.M. Tian, X.L. Fan & K.D. Hyde, Phytotaxa 205(2): 95 (2015)

F516A9B3-DA5A-527D-A8B0-C993F2B2608A

##### Descriptions.

See Fan et al. 2015.

##### Materials examined.

China • Jilin Province, Yanbian Korean Autonomous Prefecture, Yanji City, Maoershan National Forest (42°51'12.96"N, 129°28'24.06"E), alt. 297 m, on branches of *Larixolgensis*, 7, Sept, 2022, C. Peng, X.Y. Zhang (BJFC-S2374, living culture CFCC 70812, CFCC70813).

##### Notes.

**Figure 5.**
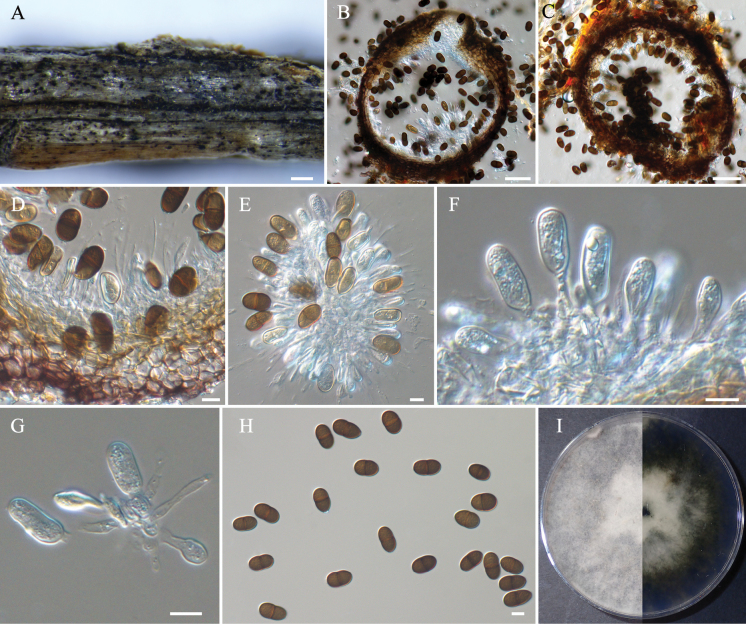
*Phaeobotryonrhois* (BJFC-S2374) **A** habit of conidiomata on twig **B** longitudinal section through a conidioma **C** transverse section of a conidioma **D–G** conidiogenous cells and conidia **H** conidia **I** colony on PDA after 7 days. Scale bars: 500 µm (**A**); 50 µm (**B, C**); 10 µm (**D–H**).

*Phaeobotryonrhois* was first discovered and reported on *Rhustyphina* (Fan et al. 2015). This species has also been isolated from diseased branches of the hosts *Dioscoreanipponica*, *Platycladusorientalis*, and *Rhamnusdavurica* (Pan et al. 2019), as well as from Populusalbavar.pyramidalis (Lin et al. 2023). Morphologically, its asexual morph (Fig. 5) aligns with the description provided by Fan et al. (2015). Phylogenetically, the isolates CFCC 70812 and CFCC 70813 clustered within a clade alongside *P.rhois*, demonstrating high statistical support (MP/ML/BI = 96/99/0.99) (Fig. 1). The current study expands its host range to include *L.olgensis*.

## ﻿Discussion

This paper describes and illustrates four species of *Phaeobotryon* from China. These species included two new species, namely *P.laricinum* and *P.longiparaphysium*, and two new host records, *P.aplosporum* and *P.rhois* from *L.olgensis*. This is the first time that this genus has been discovered from *Larix*.

Phillips et al. (2008) summarized the characteristics of *Phaeobotryon* as 2-septate, brown ascospores with an apiculus at each end. However, among 13 species of *Phaeobotryon*, only *P.mamane* and *P.quercicola* have both sexual and asexual morphs described so far, while only the sexual morphs of *P.cupressi* are documented. The remaining ten species have only asexual morphs described (Phillips et al. 2005, 2008; Abdollahzadeh et al. 2009; Fan et al. 2015; Daranagama et al. 2016; Zhu et al. 2018; Pan et al. 2019; Wijayawardene et al. 2021; Zhang et al. 2021; Lin et al. 2023; Peng et al. 2023; Wu et al. 2024). Therefore, recent papers have distinguished between species of *Phaeobotryon* by asexual morphological characteristics, such as the conidial septation and conidial size (Daranagama et al. 2016; Zhu et al. 2018; Pan et al. 2019; Zhang et al. 2021; Peng et al. 2023; Wu et al. 2024). However, there are many similarities in conidial morphology, with 1(–2) septate or aseptate conidia, similar pigmentation variations, and some species have overlapping conidial sizes. For example, *P.juniperi* and *P.platycladi* have overlapping sizes of conidia (Table 3). So, the combination of morphology and phylogenetics is essential for further clarifying the affinities between species in academic research.

Numerous reports have documented the presence of *Phaeobotryon* on diseased plants (Zhu et al. 2018; Wijayawardene et al. 2021; Zhang et al. 2021; Lin et al. 2023; Peng et al. 2023; Wu et al. 2024), with *P.negundinis* identified as a causal agent of branch blight in *Malus* spp. (Evgeny and Walid 2023). However, some studies do not include inoculation experiments necessary to definitively establish the pathogenicity of these species. Similarly, the four *Phaeobotryon* species described in this paper were found on dead and dying *Larix* branches; their pathogenicity, however, requires confirmation through further inoculation experiments. Future research should focus on elucidating the pathogenicity of *Phaeobotryon* while exploring the diversity of its species.

## Supplementary Material

XML Treatment for Phaeobotryon laricinum

XML Treatment for Phaeobotryon longiparaphysium

XML Treatment for Phaeobotryon aplosporum

XML Treatment for Phaeobotryon rhois

## ﻿Additional information

### Author ORCIDs

Yeting Zhu https://orcid.org/0009-0004-6613-1561

Yingmei Liang https://orcid.org/0000-0002-1690-5512

Cheng Peng https://orcid.org/0009-0005-9619-8246

### Data availability

All of the data that support the findings of this study are available in the main text.
